# The role of plasma exchange in treating post-transplant focal segmental glomerulosclerosis: A systematic review and meta-analysis of 77 case-reports and case-series

**DOI:** 10.1186/s12882-016-0322-7

**Published:** 2016-07-29

**Authors:** Abdullah Kashgary, Jessica M. Sontrop, Lihua Li, Ahmed A. Al-Jaishi, Zainab N. Habibullah, Roaa Alsolaimani, William F. Clark

**Affiliations:** 1 Division of Nephrology, Department of Medicine, Western University, London, Canada; 2 Department of Medicine, King Abdulaziz University Hospital, King Abdulaziz University, Jeddah, Kingdom of Saudi Arabia; 3 Department of Epidemiology and Biostatistics, Western University, London, Canada; 4 Kidney Clinical Research Unit, London Health Sciences Centre, 339 Windermere Road, London, ON Canada N6A 5A5; 5 Victoria Hospital, 800 Commissioners Road East, A2-343, London, ON Canada N6A 5W9

**Keywords:** Focal segmental glomerulosclerosis, Kidney transplantation, Plasma exchange, Plasmapheresis, Systematic review

## Abstract

**Background:**

Evidence on the role of plasma exchange for treating recurrent post-transplant focal segmental glomerulosclerosis (FSGS) comes largely from individual cases and uncontrolled series. We conducted a systematic review and meta-analysis to estimate the remission rate after treatment with plasma exchange, and to determine if remission varied with patient or treatment characteristics.

**Methods:**

We searched MEDLINE, EMBASE, Science Citation Index Expanded, and the Conference Proceedings Citation Index (Science and BIOSIS) for studies of patients with post-transplant recurrent FSGS who were treated with plasma exchange after recurrence (1950–2012). Of 678 studies screened, 77 met our inclusion criteria: 34 case reports (45 patients) and 43 case series (378 patients). We extracted patient-level data from each study and used random-effects models to calculate remission, defined as proteinuria <3.5 g/day (partial) or <0.5 g/day (complete).

**Results:**

The overall remission rate in 423 patients with outcome data was 71 % (95 % CI: 66 % to 75 %). In 235 patients with data on age, remission was similar for adults and children: 69.1 % (95 % CI: 59.6 % to 77.2 %) and 70.2 % (95 % CI: 61.1 % to 77.9 %). Males were more likely to achieve remission (OR = 2.85; 95 % CI: 1.44 to 5.62) and patients treated within 2 weeks of recurrence showed a trend towards higher likelihood of remission (OR = 2.16; 95 % CI: 0.93 to 5.01). Proteinuria >7 g/day at recurrence was inversely associated with remission (OR = 0.43; 95 % CI: 0.19 to 0.97). Age and type of kidney transplant (living vs. deceased) did not associate with remission.

**Conclusion:**

In this systematic review of patients with recurrent post-transplant FSGS, 71 % of patients achieved full or partial remission after treatment with plasma exchange; however, extensive missing data and lack of a control group limit any conclusions on causality.

**Electronic supplementary material:**

The online version of this article (doi:10.1186/s12882-016-0322-7) contains supplementary material, which is available to authorized users.

## Background

Focal segmental glomerulosclerosis (FSGS) is the most common acquired cause of kidney failure in children (after hereditary causes), and accounts for nearly 40 % of cases of nephrotic syndrome in adults [1, 2]. FSGS is a clinical-pathological syndrome characterized by scarred glomeruli, excessive protein excretion, and injured epithelial podocytes. The primary cause is unknown in approximately 80 % of cases [2] and available treatments have limited effectiveness. Even with treatment, 30–60 % of patients progress to kidney failure within 5–10 years and among those who receive a kidney transplant, severe proteinuria recurs in 30–55 % of patients, often within hours or days of grafting [3–7].

Recent studies suggest that primary FSGS may be caused by plasma-borne factors that increase glomerular permeability to albumin [3]. Treatment with plasma exchange became a logical step when several subtypes of FSGS were found to recur in allograft-transplanted kidneys, and with the discovery of potential pathological circulating factors [7–10]. Plasma exchange replaces the patient's blood plasma with a donor plasma product, removing potential pathological factors from the patient’s circulation, and has proved effective for treating acute autoimmune disorders such as Guillain-Barre syndrome and chronic conditions such as myasthenia gravis [11, 12]. While current guidelines support the use of plasma exchange for recurrent post-transplant FSGS, evidence on treatment efficacy comes largely from case reports and uncontrolled case series [3, 13]. We conducted a systematic review of all relevant studies of patients with post-transplant recurrent FSGS (both adults and children) who were treated with plasma exchange after recurrence. We extracted patient-level data from these studies, and estimated the rate of remission after treatment (defined as proteinuria <3.5 g/day (partial) or <0.5 g/day (complete)), and examined if remission varied with patient or treatment characteristics.

## Methods

Following the Preferred Reporting Items for Systematic Reviews and Meta-Analysis (PRISMA) and Meta-Analysis of Observational Studies in Epidemiology (MOOSE) guidelines [14, 15], we performed a systematic review of English-language articles using MEDLINE and PreMedline (OVID, 1966 to December 2012), EMBASE (1979 to December 2012), Science Citation Index Expanded (1945 to December 2012), Conference Proceedings Citation Index-Science (1990 to December 2012), BIOSIS Previews (1955 to December 2012). Additional studies were identified through reviewing reference lists of retrieved studies. A detailed search strategy is provided in Appendix I. The study is exempt from ethics approval because we synthesized data from previous published studies.

Study eligibility criteria were determined a priori. We included all study designs of patients identified as having recurrent post-renal transplant focal glomerulosclerosis (FSGS) who were treated with plasma exchange at the time of recurrence. Studies with no data on post-treatment remission (defined below) were excluded. No randomized controlled trials were identified in our search. While some studies contained a comparison group of patients with recurrent post-transplant FSGS who did *not* receive plasma exchange, these comparison groups largely comprised historical controls treated in the decade prior to the cases. Data from these controls were not included since these patients would have lacked access to the same supportive treatment as cases (leading to biased comparisons favoring plasma exchange). We defined a study as a case report if it described one or two patients with recurrent FSGS who were treated with plasma exchange. A study was defined as a case series if it described three or more patients with recurrent FSGS who were treated with plasma exchange (followed prospectively or retrospectively; the largest sample size was 23).

The primary outcome in our study, remission after treatment with plasma exchange, was defined as proteinuria <0.5 g/day (complete remission) or proteinuria between 0.5 − 3.5 g/day (partial remission). To be conservative, if data on protein was not available, but the patient was reported as having achieved remission in the primary study, we coded the patient as achieving partial rather than complete remission. In the final analysis, patients who achieved complete or partial remission were defined as *responders* and those who did not achieve remission were defined as *non-responders*.

### Data extraction

A data extraction form was prepared and piloted to determine if changes were required before extracting data from the full review. Two review authors (AK and RA) independently extracted patient-level data from included articles using pre-defined criteria and compared data to achieve maximum reliability. Any disagreements were reviewed and resolved by the authors.

Information was extracted on study and patient characteristics, and on treatment details and outcomes. Study characteristics included year of publication, country, study design, number of participants, and median follow-up time. Patient characteristics included age, sex, type of kidney transplant (living or deceased), level of proteinuria at time of FSGS recurrence, biopsy testing, time to recurrence, and time from recurrence to treatment with plasma exchange. Treatment details included the number of plasma exchange sessions, the number of treatments, and the use of rituximab or steroids. Data on patient outcomes at the end of follow-up included proteinuria, estimated glomerular filtration rate (eGFR), progression to end-stage renal disease (ESRD), and the time from transplant to ESRD.

Availability of Data and Materials: All the data supporting the conclusions of this article are contained within the manuscript. The individual patient-level dataset was not made publically available due to containing potentially identifying patient data; however, the data may be made available from the authors upon request.

### Analysis

Where possible, we collected individual-level data from each study; however, missing data was >50 % for many patient characteristics. Restricting the sample to only those patients with complete data on prognostic variables would have excluded 70 % of the available sample, possibly introducing selection and reporting biases. For this reason, we present all available data, specifying the amount of missing data for each variable (summarized in Table 1 and in the text of the results). We used random-effects models to estimate remission, which incorporates study-specific random effects in addition to patient-specific effects. We report the remission rate in the overall sample and in sub-groups stratified by study design and age.

**Table 1:** Characteristics and outcomes of patients treated with plasma exchange after primary post-renal transplant focal segmental glomerulosclerosis (1950–2012)

	Median or n (% of available data)	Data not reported n (% of 423)
Characteristics at time of recurrence		
Age at time of treatment (years), median (IQR)	17 (12,33)	188 (44 %)
< 18 years, n (%)	119/235 (50.6 %)	
Gender, n (%)		211 (50 %)
Male	126/212 (59.4 %)	
Female	86/212 (40.6 %)	
Time to recurrence (days), median (IQR)	4 (1,18)	131 (31 %)
Proteinuria (g/day), median (IQR)	7 (5,12)	275 (65 %)
Biopsy conducted, n (%)	211/266 (79.3 %)	157 (37 %)
Kidney transplant type, n (%)		248 (59 %)
Living donor	62/175 (35.4 %)	
Deceased donor	113/175 (64.6 %)	
Treatment characteristics		
Time from recurrence to start of plasma exchange (days), median (IQR)	1 (0,3)	193 (46 %)
Delayed treatment start, n (%)		193 (46 %)
More than two weeks	28/230 (12.2 %)	
More than one month	22/230 (9.6 %)	
Total number of plasma exchange sessions, median (IQR)	12 (10,20)	191 (45 %)
Total weeks of plasma exchange treatment, median (IQR)	12 (4,28)	309 (73 %)
Received rituximab	41/404 (4.7 %)	19 (4 %)
Received steroids	341/344 (99.1 %)	79 (19 %)
Post-treatment outcomes		
Remission, n (%)	317/423 (74.0 %)	0
Complete remission (proteinuria <0.5 g/day)	198/423 (46.8 %)	
Partial remission (proteinuria: 0.5–3.5 g/day)^a^	119/423 (28.1 %)	
eGFR at follow up (ml/min/1.73 m^2^), median (IQR)	44 (25,74)	288 (68 %)
Proteinuria at follow-up (g/day), median (IQR)	0.2 (0,2)	259 (61 %)
End-stage renal disease, n (%)	83/381 (21.8 %)	42 (10 %)
Time to ESRD after transplant (years), median (IQR)	0.9 (0.3,2.0)	383 (91 %)

*Abbreviations*: *eGFR* estimated glomerular filtration rate, *ESRD* end-stage renal disease, *IQR* inter-quartile range

^a^If protein data were not reported in the primary study, but the patient was reported as having achieved remission, we coded the patient as a partial remission

Characteristics of responders (those who achieved full or partial remission) and non-responders were first summarized using simple descriptive statistics. Percentages were calculated based on valid denominators (i.e. denominators exclude patients with missing data on the characteristic of interest). Continuous data are summarized using medians and interquartile ranges (IQR: 25th percentile, 75th percentile). We used then generalized estimating equations (adjusted for within-study clustering) to examine associations between remission and several prognostic variables: age (<18 years and ≥18 years), sex, type of kidney transplant (living vs. deceased), median proteinuria at recurrence (<7 g/day vs. ≥7 mg/d), delayed treatment start (≤2 weeks vs. >2 weeks), and treatment with rituximab. Odds ratios (OR) and 95 % confidence intervals (95 % CI) are reported to describe the statistical uncertainty of effect estimates [16]. All data were analyzed using SAS 9.3 (SAS Institute Inc., Cary, NC, USA.).

## Results

### Study and patient characteristics

**Fig. 1:**
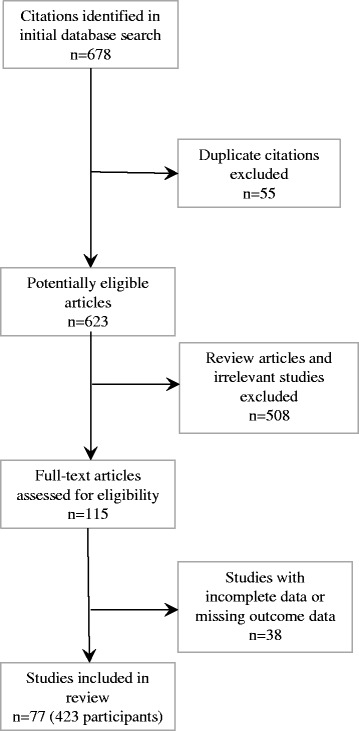
Flow diagram of included studies (references for 77 included studies and 38 excluded studies are provided in Additional file 1)

Of 678 citations identified in our search, 77 met our inclusion criteria (Fig. 1; references provided in Additional file 1): 34 were case reports (45 patients) and 43 were case series [378 patients]. Of 77 studies, 58 (76 %) were full-text publications (229 patients) and 18 (24 %) were abstracts (124 patients). Studies were conducted in 21 countries between 1985 and 2012. The median follow-up time was 19 months (IQR 9, 56).

Characteristics of 423 patients with recurrent post-transplant FSGS are summarized in Table 1. Raw data are presented along with the percentage of missing data for each characteristic. The median time to FSGS recurrence was 4 days (IQR 1, 18) (reported for 135 patients). Median age at recurrence was 17 years (IQR 12, 33) (reported for 235); 51 % were younger than 18 years (119/235) and 59 % were male (126/212). Median proteinuria at recurrence was 7 g/day (IQR 5, 12) (reported for 148). The median time from recurrence to treatment with plasma exchange was 1 day (IQR 0, 3); however, 12 % (28/230) did not receive plasma exchange until >2 weeks after recurrence. The total number of plasma exchange sessions ranged from 1 to 200 with a median of 12 sessions (IQR 10, 20). In addition to treatment with plasma exchange, 10 % received rituximab (41/404) and 99 % received steroids (341/344).

### Remission from proteinuria after treatment with plasma exchange

As shown in Table 1, 198 of 423 patients (46.8 %) achieved complete remission after treatment with plasma exchange, and 119/423 (28.1 %) achieved partial remission. In a random-effects model that accounted for patient- and study-specific random effects, the overall remission rate was 71.0 % (95 % CI: 66.2 % to 75.4 %). The remission rate in case-series studies was 72.5 % (95 % CI: 67.4 % to 77.1 %) compared with 63.4 % in case reports (95 % CI: 50.3 % to 74.8 %). In a restricted sample of 235 patient with data on age, remission was similar for adults and children: 69.1 % (95 % CI: 59.6 % to 77.2 %) and 70.2 % (95 % CI: 61.1 % to 77.9 %), respectively.

A descriptive comparison of responders (those who achieved full or partial remission) and non-responders is shown in Table 2. Responders appeared to have a shorter time to recurrence (3 days vs. 7 days) and lower proteinuria at recurrence (7 g/day vs. 9 g/day); however, data on protein was missing for 65 % of patients. The median time to plasma exchange treatment was 1 day in both groups; however, delayed treatment start (>2 weeks) appeared to be more common among non-responders (21 % vs. 9 %), and 19 % of non-responders had not received plasma exchange within one month of recurrence, compared with only 6 % of responders. Most other variables were similar between groups, including age (median 17 years), type of kidney transplant (deceased donor: 64 %), and the median number of plasma exchange sessions received (12 sessions). Finally, 9 % of responders received rituximab compared with 14 % of non-responders. Of the 41 patients who received rituximab, 29 (70.7 %) achieved remission, 7 responded to rituximab but not plasma exchange, and 7 did not respond to either plasma exchange or rituximab. After a median follow-up of 19 months (IQR 9, 56), median proteinuria was 0.1 g/day among responders and 4 g/day among non-responders, and the median eGFR was 60 ml/min/1.73 m^2^ (IQR 37, 84) and 15 ml/min/1.73 m^2^ (15, 40), respectively; 11 % of responders developed end-stage kidney disease compared with 57 % of non-responders.

**Table 2:** Remission after treatment with plasma exchange for recurrent post-transplant FSGS: a descriptive comparison using all available data

	Remission	
	Yes^a^	No
Pre-treatment characteristics		
Age at time of treatment (years), median (IQR)	17 (13,32)	17 (11,38)
< 18 years, n (%)	89 (50.6 %)	30 (50.8 %)
Male, n (%)	106 (65.8 %)	20 (39.2 %)
Pre-treatment proteinuria (g/day), median (IQR)	7 (5,12)	9 (6,15)
Biopsy conducted at time of recurrence, n (%)	157 (77.3 %)	54 (85.7 %)
Time to recurrence (days), median (IQR)	3 (1,14)	7 (1,60)
Kidney transplant type, n (%)		
Living donor transplant	47 (35.1 %)	15 (36.6 %)
Deceased donor transplant	87 (64.9 %)	26 (63.4 %)
Treatment characteristics		
Time from recurrence to start of plasma exchange (days), median (IQR)	1 (0,2)	1 (0,7)
Delayed treatment start, n (%)		
More than two weeks	15 (9.0 %)	13 (20.6 %)
More than one month	10 (6.0 %)	12 (19.1 %)
Total number of plasma exchange sessions, median (IQR)	12 (10,20)	12 (9,20)
Received rituximab	27 (8.9 %)	14 (14.0 %)
Received steroids	250 (99.2 %)	86 (98.9 %)
Post-treatment outcomes		
eGFR at follow up (ml/min/1.73 m^2^), median (IQR)	60 (37,84)	15 (15,40)
Proteinuria at follow-up (g/day), median (IQR)	0.1 (0,0.5)	4 (1,6)
End-stage renal disease (ESRD), n (%)	31 (10.7 %)	52 (57.1 %)
Time to ESRD after transplant (years), median (IQR)	1.4 (0.3,2.0)	0.8 (0.3,2.0)

*Abbreviations*: *eGFR* estimated glomerular filtration rate, *ESRD* end-stage renal disease, *IQR* inter-quartile range (25th percentile, 75th percentile)

^a^Complete or partial remission: Full remission was defined as proteinuria <0.5 g/day; partial remission was defined as proteinuria between 0.5 and 3.5 g/day. If protein data were not reported in the primary study, but the patient was reported as having achieved remission, we coded the patient as a partial remission

The results of the statistical analysis were consistent with the descriptive analysis. In univariable regression models that accounted for within-study clustering (Table 3), males were nearly three times as likely to achieve remission as females (OR 2.85; 95 % CI: 1.44 to 5.62), and patients who received plasma exchange within two weeks of recurrence were twice as likely to achieve remission as patients whose treatment initiation was delayed (OR 2.16; 95 % CI: 0.93 to 5.01), although the latter effect estimate did not reach statistical significance. Patients with proteinuria >7 g/day at recurrence were less likely to achieve remission (OR 0.43; 95 % CI: 0.19 to 0.97). Age, type of kidney transplant, and treatment with rituximab were not statistically associated with remission. Effect sizes remained similar when we restricted the sample to 110 patients with complete data on age, gender, and time to treatment with plasma exchange, although confidence intervals became wider and only gender remained statistically significant.

**Table 3:** Associations with remission: Individual-level meta-regression of available data

	OR (95 % CI)
Adults vs. children (235 patients; 55 studies)	1.09 (0.55 to 2.16)
Males vs. females (212 patients; 54 studies)	2.85 (1.44 to 5.62)
Proteinuria at recurrence (≥7 g/day vs. <7 g/day) (148 patients; 40 studies)	0.43 (0.19 to 0.97)
Received rituximab (404 patients; 72 studies)	0.60 (0.21 to 1.75)
Transplant type (living vs. deceased) (423 patients; 46 studies)	1.00 (0.43 to 2.30)
Plasma exchange within 2 weeks of FSGS recurrence (230 patients; 54 studies)	2.16 (0.93 to 5.01)

Odds ratios (OR) and 95 % confidence intervals estimated using generalized linear estimating equations to account for within-study clustering

## Discussion

We conducted a systematic review of 77 studies of patients with post-transplant FSGS who were treated with plasma exchange at the time of recurrence. Overall, 71 % of patients achieved complete or partial remission from proteinuria after treatment with plasma exchange (95 % CI: 66 % to 75 %). Males were more likely to achieve remission than females, and patients who received plasma exchange within two weeks of recurrence appeared more likely to achieve remission than patients whose treatment initiation was delayed, although the latter did not reach statistical significance (the 95 % confidence interval spanned 0.9 to 5.0). Patients with higher proteinuria at recurrence (>7 g/day) were less likely to achieve remission. Age and type of kidney transplant (living vs. deceased) were not associated with remission.

While several narrative reviews have examined the role of plasma exchange for recurrent post-transplant FSGS [3, 6, 7, 17, 18], our study is the first systematic review of this literature. We identified 34 case reports and 43 case series (76 % were full-text publications and 24 % were abstracts). Although patient-level data was missing for many prognostic variables including age and sex, we were able to extract individual-level data on remission for 423 patients. Overall 71 % of patients with recurrent post-transplant FSGS achieved complete or partial remission from proteinuria after treatment with plasma exchange. This estimate is consistent with rates described in narrative reviews (60 % to 73 %), and appears favorable when compared to historical controls (i.e. patients with recurrent post-transplant FSGS not treated with plasma exchange), where reported remission rates are less than 30 %; however, most historical controls would have lacked access to modern supportive therapy and immunosuppressive treatment [19, 20].

In a descriptive comparison of responders (those who achieved remission) and non-responders, we noted that responders appeared to have a shorter time to FSGS recurrence than non-responders (3 days vs. 7 days), and lower levels of proteinuria at recurrence (7 g/day vs. 9 g/day). While this may reflect differences in disease phenotype, progression, or severity, it is also possible that responders were monitored more closely after transplantation with the result that recurrent FSGS was detected earlier and treated more quickly. In regression analysis, patients who received plasma exchange within two weeks of recurrence appeared more likely to achieve remission than patients whose treatment initiation was delayed. Although there is uncertainty about the size of this effect (the 95 % CI spanned 0.9 to 5.0; n = 230), these data suggest a beneficial effect of prompt treatment with plasma exchange (i.e. a harmful effect seems unlikely). However, the observational nature of the data limits our ability to make any causal inferences about the efficacy of plasma exchange independent of other concomitant or supportive therapies.

Proteinuria may herald recurrent FSGS even if an early biopsy does not show glomerular abnormalities [17]. KDIGO guidelines on proteinuria screening for post-transplant primary FSGS recommend daily testing for one week, weekly testing for four weeks, and every three months thereafter; however, this schedule could result in delayed detection and treatment among those who relapse more than seven days after transplantation (who may not then receive treatment until >14 days after recurrence, when it may be too late). In some cases, delayed treatment may result from confusion about recurrent patients mislabeled as suffering from prior minimal lesion nephropathy. However, the variability in the literature suggests that early observation and intervention strategies were not a practice standard, and current guidelines may need to extend the period of initial proteinuria testing.

In our study, males were nearly three times more likely to achieve remission than females. At present, it is difficult to speculate on this marked gender variability in response to plasma exchange therapy; however, other studies have noted an increased incidence of recurrent FSGS in females compared with males [6, 21]. Of note, we saw similar remission rates for recipients of kidneys from living vs. deceased donors, which may be encouraging given that other studies have noted an increased risk of FSGS recurrence among recipients of kidneys from living vs. deceased donors [12, 22].

Our findings should be interpreted with caution given the many limitations of these data. All studies in our review were observational with no concurrent comparison groups. Missing data was greater than 50 % for many patient characteristics and prognostic variables, and eligibility criteria were not always reported, limiting the assessment of selection bias and external validity. Further, treatment protocols were not standardized across patients or studies. While all patients in this review received plasma exchange therapy for post-transplant recurrent FSGS, many studies did not report the type of replacement fluid used (i.e. 5 % serum albumin solution vs. fresh frozen plasma). The time between recurrence and treatment varied considerably as did the number of plasma exchange sessions received. As well, immunosuppression treatment, pre-transplant prophylactic plasma exchange, follow-up time, and outcome assessment varied widely across patients and studies.

Estimating the overall effect of these biases is difficult. While our estimate of remission may be inflated by selection bias and publication bias, it may also be attenuated by the wide variation in treatment protocols across studies. For example, in one study that used a standardized protocol to treat 10 consecutive patients with recurrent FSGS, nine patients achieved complete remission and one achieved partial remission when plasma exchange was initiated within ten days of recurrence and administered three times per week for three weeks (tapered to once per month after five months) along with high-dose oral steroids and intravenous cyclosporine. Unfortunately, a parallel-group randomized controlled trial may not be feasible given the rareness of this condition (<1 %), and also, it is questionable whether sufficient equipoise for such a trial exists.

Future research should examine and refine the treatment protocol (timing, dosage, prescription of 5 % serum albumin solution vs. fresh frozen plasma, duration of plasma exchange treatment after remission); long-term patient outcomes (in some cases FSGS may recur months or years after transplant); [8] the efficacy of pre-transplant plasma exchange for preventing recurrence; supportive therapy; and develop new hypothesis-driven therapies—a difficult endeavor given that the cause of primary FSGS remains unknown. While many studies support a plasma-borne pathophysiology, a specific mechanism has yet to be identified despite intensive research, and we still lack a reliable diagnostic test [3, 17].

## Conclusions

In this systematic review of patients with recurrent post-transplant FSGS, 71 % of patients achieved full or partial remission from proteinuria after treatment with plasma exchange. Patients treated within two weeks of recurrence appeared to have a higher likelihood of remission from proteinuria, although this finding must be interpreted with caution given the observational nature of the data. Nonetheless, it seems prudent to support careful observation in the post-transplant period (daily proteinuria testing for at least 14 days) for patients with a prior diagnosis of FSGS or minimal change nephrotic syndrome; if proteinuria is detected, immediate initiation of plasma exchange therapy may be a potentially useful therapeutic option.

## Abbreviations

CI, confidence intervals; eGFR, estimated glomerular filtration rate; ESRD, end stage renal disease; FSGS, focal segmental glomerulosclerosis; IQR, interquartile range; KDIGO, kidney disease improving global outcomes; MOOSE, meta-analysis of observational studies in epidemiology; OR, odds ratio; PRISMA, preferred reporting items for systematic reviews and meta-analysis

## Additional file

Additional file 1: Citations of Included and Excluded Studies. (PDF 121 kb)

## Appendix I

### Search Strategy

A systematic literature search was performed for all relevant English and non-English language articles using MEDLINE and PreMedline (OVID, 1966 to December 2012), EMBASE (1979 to December 2012), Science Citation Index Expanded (1945 to December 2012), Conference Proceedings Citation Index- Science (1990 to December 2012), BIOSIS Previews (1955 to December 2012). Additional studies were identified by reviewing reference lists of retrieved studies and by contacting clinical experts. Search terms included plasmapheresis, aphaeresis, plasma exchange, focal segmental glomerulosclerosis, FSGS, focal glomerulosclerosis, recurrent FSGS

## References

[1] United States Renal Data System. Chapter 8: Pediatric ESRD. 2012:295–308. http://www.usrds.org/2012/pdf/v2_ch8_12.pdf. Accessed May 18, 2015.

[2] D’Agati VD, Kaskel FJ, Falk RJ (2011). Focal segmental glomerulosclerosis. N Engl J Med.

[3] Cravedi P, Kopp JB, Remuzzi G (2013). Recent progress in the pathophysiology and treatment of FSGS recurrence. Am J Transpl.

[4] Artero M, Biava C, Amend W, Tomlanovich S, Vincenti F (1992). Recurrent focal glomerulosclerosis: natural history and response to therapy. Am J Med.

[5] Trachtman R, Sran SS, Trachtman H. Recurrent focal segmental glomerulosclerosis after kidney transplantation. Pediatr Nephrol. 2015;30:1793–1802. 10.1007/s00467-015-3062-1 25690943

[6] Straatmann C, Kallash M, Killackey M (2014). Success with plasmapheresis treatment for recurrent focal segmental glomerulosclerosis in pediatric renal transplant recipients. Pediatr Transplant.

[7] Vinai M, Waber P, Seikaly MG (2010). Recurrence of focal segmental glomerulosclerosis in renal allograft: an in-depth review. Pediatr Transpl.

[8] Ponticelli C, Glassock RJ (2010). Posttransplant recurrence of primary glomerulonephritis. Clin J Am Soc Nephrol.

[9] Carraro M, Caridi G, Bruschi M (2002). Serum glomerular permeability activity in patients with podocin mutations (NPHS2) and steroid-resistant nephrotic syndrome. J Am Soc Nephrol.

[10] Sharma M, Sharma R, McCarthy ET, Savin VJ (2004). The focal segmental glomerulosclerosis permeability factor: biochemical characteristics and biological effects. Exp Biol Med.

[11] Raphaël JC, Chevret S, Hughes RA, Annane D (2002). Plasma exchange for Guillain-Barré syndrome. Cochrane Database Syst Rev.

[12] First MR (1995). Living-related donor transplants should be performed with caution in patients with focal segmental glomerulosclerosis. Pediatr Nephrol.

[13] Kidney Disease: Improving Global Outcomes (KDIGO) Transplant Work, Group (2009). KDIGO clinical practice guideline for the care of kidney transplant recipients. Am J Transplant.

[14] Stewart LA, Clarke M, Rovers M (2015). Preferred Reporting Items for a Systematic Review and Meta-analysis of Individual Participant Data. JAMA.

[15] Stroup DF, Berlin JA, Morton SC (2000). Meta-analysis of observational studies in epidemiology: a proposal for reporting. Meta-analysis Of Observational Studies in Epidemiology (MOOSE) group. JAMA.

[16] Gardner MJ, Altman DG (1988). Estimating with confidence. Br Med J (Clin Res Ed).

[17] Ponticelli C (2010). Recurrence of focal segmental glomerular sclerosis (FSGS) after renal transplantation. Nephrol Dial Transplant.

[18] Davenport RD (2001). Apheresis treatment of recurrent focal segmental glomerulosclerosis after kidney transplantation: re-analysis of published case-reports and case-series. J Clin Apher.

[19] Canaud G, Zuber J, Sberro R (2009). Intensive and prolonged treatment of focal and segmental glomerulosclerosis recurrence in adult kidney transplant recipients: a pilot study. Am J Transplant.

[20] Deegens JKJ, Andresdottir MB, Croockewit S, Wetzels JFM (2004). Plasma exchange improves graft survival in patients with recurrent focal glomerulosclerosis after renal transplant. Transpl Int.

[21] Sener A, Bella AJ, Nguan C, Luke PPW, House AA. Focal segmental glomerular sclerosis in renal transplant recipients: predicting early disease recurrence may prolong allograft function. Clin Transplant. 2009;23(1):96–100. 10.1111/j.1399-0012.2008.00908.x 19200221

[22] Baum MA (2004). Outcomes after renal transplantation for FSGS in children. Pediatr Transplant.

