# Supplementary material for: The role of plasma exchange in treating post-transplant focal segmental glomerulosclerosis: A systematic review and meta-analysis of 77 case-reports and case-series
Source: BMC Nephrol. 2016 Jul 29;17:104. doi: 10.1186/s12882-016-0322-7 (PMC4966699; doi:10.1186/s12882-016-0322-7)
Supplement: Additional file 1: — Citations of Included and Excluded Studies. (PDF 121 kb) [file 12882_2016_322_MOESM1_ESM.pdf]

Appendix II: Citations of Included and Excluded Studies

Citations of Included Studies

1. Adalat S. Successful Treatment of Recurrent FSGS In Second Renal Transplant. Pediatr Transpl. 2009/04/16 ed. 2009;13 Suppl 1:69.

2. Akash N. Recurrence of focal segmental glomerulosclerosis after renal transplantation: a case report. Saudi J Kidney Dis Transpl. 2007;18(1):91–4.

3. Andresdottir MB, Ajubi N, Croockewit S, Assmann KJ, Hibrands LB, Wetzels JF. Recurrent focal glomerulosclerosis: natural course and treatment with plasma exchange. Nephrol Dial Transpl. 1999/10/27 ed. 1999;14(11):2650–6.

4. Araya CE, Dharnidharka VR. The factors that may predict response to rituximab therapy in recurrent focal segmental glomerulosclerosis: a systematic review. J Transplant. 2011 Jan;2011:374213.

5. Artero ML, Sharma R, Savin VJ, Vincenti F. Plasmapheresis reduces proteinuria and serum capacity to injure glomeruli in patients with recurrent focal glomerulosclerosis. Am J Kidney Dis. 1994;23(4):574–81.

6. Baluarte HJ. Recurrence of Focal Segmental Glomerulosclerosis (FSGS) In Children After Renal Transplantation: A Single Center Experience. Pediatr Transplant. Blackwell Publishing Ltd; 2011;15:52.

7. Bayrakci US, Baskin E, Sakalli H, Karakayali H, Haberal M. Rituximab for post-transplant recurrences of FSGS. Pediatr Transpl. 2008/09/30 ed. 2009;13(2):240–3.

8. Belson A, Yorgin PD, Al-Uzri AY, Salvatierra O, Higgins J, Alexander SR. Long-term plasmapheresis and protein A column treatment of recurrent FSGS. Pediatr Nephrol. 2001;16(12):985–9.

9. Canaud G, Dion D, Zuber J, Gubler MC, Sberro R, Thervet E, et al. Recurrence of nephrotic syndrome after transplantation in a mixed population of children and adults: course of glomerular lesions and value of the Columbia classification of histological variants of focal and segmental glomerulosclerosis (FSGS). Nephrol Dial Transpl. 2009/09/24 ed. 2010;25(4):1321–8.

10. Canaud G, Zuber J, Sberro R, Royale V, Anglicheau D, Snanoudj R, et al. Intensive and prolonged treatment of focal and segmental glomerulosclerosis recurrence in adult kidney transplant recipients: a pilot study. Am J Transplant. 2009 May;9(5):1081–6.

11. Cara Fuentes G, Garcia Meseguer C, Peña Carrion A, Melgosa Hijosa M, Garcia-Pose A, Alonso Melgar A, et al. Long-term outcome of focal segmental glomerulosclerosis after pediatric renal transplantation. Pediatr Nephrol. Springer-Verlag; 2010;25(3):529–34.

12. Cheong HI, Han HW, Park HW, Ha IS, Han KS, Lee HS, et al. Early recurrent nephrotic syndrome after renal transplantation in children with focal segmental glomerulosclerosis. Nephrol Dial Transpl. 2000;15(1):78–81.

13. Cleper R. Focal Segmental Glomerulosclerosis in Pediatric Kidney Transplantation – 30 Years Experience. Pediatr Transplant. Blackwell Publishing Ltd; 2011;15:43.

14. Cochat P, Kassir A, Colon S, Glastre C, Tourniaire B, Parchoux B, et al. Recurrent nephrotic syndrome after transplantation: early treatment with plasmaphaeresis and cyclophosphamide. Pediatr Nephrol. 1993;7(1):50–4.

15. Damodar A, Mustafa R, Bhatnagar J, Panesar M, Gundroo A, Zachariah M, et al. Use of anti-CD20 antibody in the treatment of post-transplant glomerulonephritis. Clin Transpl. 2010/04/14 ed. 2011;25(3):375–9.

16. Dantal J, Baatard R, Hourmant M, Cantarovich D, Buzelin F, Soulillou JP. Recurrent nephrotic syndrome following renal transplantation in patients with focal glomerulosclerosis. A one-center study of plasma exchange effects. Transplantation. 1991;52(5):827–31.

17. Deegens JK, Andresdottir MB, Croockewit S, Wetzels JF. Plasma exchange improves graft survival in patients with recurrent focal glomerulosclerosis after renal transplant. Transpl Int. 2004/04/23 ed. 2004;17(3):151–7.

18. Deegens JKJ, Wetzels JFM. Treatment of recurrent focal glomerulosclerosis after renal transplantation: is prednisone essential to maintain a sustained remission? Transplantation. 2003 Apr 15;75(7):1080–1.

19. Deegens JKJ, Wetzels JFM. Rituximab for plasma exchange-dependent recurrent focal segmental glomerulosclerosis after renal transplantation. NDT Plus. 2008;1(2):85–8.

20. Dello Strologo L, Guzzo I, Laurenzi C, Vivarelli M, Parodi A, Barbano G, et al. Use of rituximab in focal glomerulosclerosis relapses after renal transplantation. Transplantation. 2009 Aug 15;88(3):417–20.

21. Delucchi A, Cano F, Rodriguez E, Wolff E. Focal segmental glomerulosclerosis relapse after transplantation: treatment with high cyclosporine doses and a short plasmaphaeresis course. Pediatr Nephrol. 1994;8(6):786–7.

22. Dijkman H, Smeets B, van der Laak J, Steenbergen E, Wetzels J. The parietal epithelial cell is crucially involved in human idiopathic focal segmental glomerulosclerosis. Kidney Int. 2005/09/17 ed. 2005;68(4):1562–72.

23. El-Firjani A, Hoar S, Karpinski J, Bell R, Deschenes MJ, Knoll GA. Post-transplant focal segmental glomerulosclerosis refractory to plasmapheresis and rituximab therapy. Nephrol Dial Transpl. 2007/11/06 ed. 2008;23(1):425.

24. Fencl F, Simková E, Vondrák K, Janda J, Chadimová M, Stejskal J, et al. Recurrence of nephrotic proteinuria in children with focal segmental glomerulosclerosis after renal transplantation treated with plasmapheresis and immunoadsorption: case reports. Transplant Proc. 2007 Dec;39(10):3488–90.

25. Franco A, Peres R, Anaya F, Niembro E, Valderrabano. Spontaneous remission of proteinuria in recurrent focal glomerulosclerosis. Reappraisal of plasma exchange treatment. Clin Nephrol. 1987;28(3):158.

26. Garcia CD, Bittencourt VB, Tumelero A, Antonello JS, Malheiros D, Garcia VD. Plasmapheresis for recurrent posttransplant focal segmental glomerulosclerosis. Transpl Proc. 2006;38(6):1904–5.

27. Gohh RY, Yango AF, Morrissey PE, Monaco AP, Gautam A, Sharma M, et al. Preemptive plasmapheresis and recurrence of FSGS in high-risk renal transplant recipients. Am J Transpl. 2005;5(12):2907–12.

28. Gonzalez E, Ettenger R, Rianthavorn P, Tsai E, Malekzadeh M. Preemptive plasmapheresis and recurrence of focal segmental glomerulosclerosis in pediatric renal transplantation. Pediatr Transplant. Blackwell Publishing Ltd; 2011;15(5):495–501.

29. Gossmann J, Scheuermann EH, Porubsky S, Kachel HG, Geiger H, Hauser IA. Abrogation of nephrotic proteinuria by rituximab treatment in a renal transplant patient with relapsed focal segmental glomerulosclerosis. Transpl Int. 2007;20(6):558–62.

30. Greenstein SM, Delrio M, Ong E, Feuerstein D, Schechner R, Kim D, et al. Plasmapheresis treatment for recurrent focal sclerosis in pediatric renal allografts. Pediatr Nephrol. 2000;14(12):1061–5.

31. Grenda R, Jarmuzek W, Piatosa B, Rubik J. Long-term effect of rituximab in maintaining remission of recurrent and plasmapheresis-dependent nephrotic syndrome post-renal transplantation - case report. Pediatr Transpl. 2010/03/25 ed. 2011;15(6):E121–5.

32. Gusmano R, Mazzucco G, Monga G, Ghiggeri GM. Focal and segmental glomerulosclerosis (FSGS). Clinical, morphological and genetic features. J Nephrol. 2004/05/21 ed. 2004;17(1):139–57.

33. Haffner K, Zimmerhackl LB, von Schnakenburg C, Brandis M, Pohl M. Complete remission of post-transplant FSGS recurrence by long-term plasmapheresis. Pediatr Nephrol. 2005;20(7):994–7.

34. Hristea D, Hadaya K, Marangon N, Buhler L, Villard J, Morel P, et al. Successful treatment of recurrent focal segmental glomerulosclerosis after kidney transplantation by plasmapheresis and rituximab. Transpl Int. 2007;20(1):102–5.

35. Hwang JH, Han SS, Huh W, Park S-K, Joo DJ, Kim MS, et al. Outcome of kidney allograft in patients with adulthood-onset focal segmental glomerulosclerosis: comparison with childhood-onset FSGS. Nephrol Dial Transplant. 2012;27(6):2559–65.

36. Iguchi Y, Tanabe K, Yagisawa T, Fuchinoue S, Kawai T, Kawaguchi H, et al. Plasmapheresis for prevention of recurrent focal segmental glomerulosclerosis of kidney allograft in adult recipients. Ther Apher. 1997;1(2):191–4.

37. Kamar N, Faguer S, Esposito L, Guitard J, Nogier MB, Durand D, et al. Treatment of focal segmental glomerular sclerosis with rituximab: 2 case reports. Clin Nephrol. 2007;67(4):250–4.

38. Kawaguchi H, Hattori M, Ito K, Takahashi K, Ota K. Recurrence of focal glomerulosclerosis of allografts in children: the efficacy of intensive plasma exchange therapy before and after renal transplantation. Transpl Proc. 1994;26(1):7–8.

39. Kent, P; Drew, M; Galacki, D; Humphreys, D; Jabs, K; Harmon, W; Kevy S. Intensive plasma exchange in children with recurrent focal segmental glomerulosclerosis post renal transplant. Artificial Organs. 1994. p. 18(2): 135.

40. Laufer J, Ettenger RB, Ho WG, Cohen AH, Marik JL, Fine RN. Plasma exchange for recurrent nephrotic syndrome following renal transplantation. Transplantation. 1988;46(4):540–2.

41. Lee, SE; Han, KH; Jung, YH; Lee, HK; Kang, HG; Ha, IS; Choi, Y; Cheong H. Clinical course of pediatric focal segmental glomerulosclerosis after renal transplant: Korean experience. Pediatr Nephrol. 2010;25(9):1891.

42. Li PK, Lai FM, Leung CB, Lui SF, Wang A, Lai KN. Plasma exchange in the treatment of early recurrent focal glomerulosclerosis after renal transplantation. Report and review. Am J Nephrol. 1993;13(4):289–92.

43. Mahesh S, Del Rio M, Feuerstein D, Greenstein S, Schechner R, Tellis V, et al. Demographics and response to therapeutic plasma exchange in pediatric renal transplantation for focal glomerulosclerosis: A single center experience. Pediatr Transplant. Blackwell Publishing Ltd; 2008;12(6):682–8.

44. Marcen R, Navarro JF, Mampaso F, Orofino L, Gonzalo A, Teruel JL, et al. Recurrence of focal-segmental glomerulosclerosis in kidney transplant patients on ciclosporin. Nephron. 1994;68(4):497–9.

45. Masutani K, Katafuchi R, Ikeda H, Yamamoto H, Motoyama K, Sugitani A, et al. Recurrent nephrotic syndrome after living-related renal transplantation resistant to plasma exchange: report of two cases. Clin Transpl. 2005;19 Suppl 1:59–64.

46. Matalon A, Markowitz GS, Joseph RE, Cohen DJ, Saal SD, Kaplan B, et al. Plasmapheresis treatment of recurrent FSGS in adult renal transplant recipients. Clin Nephrol. 2001;56(4):271–8.

47. Moriconi L, Lenti C, Puccini R, Pasquariello A, Rindi P, Batini V, et al. Proteinuria in focal segmental glomerulosclerosis: role of circulating factors and therapeutic approach. Ren Fail. 2001;23(3-4):533–41.

48. Mowry J, Marik J, Cohen A, Hogg R, Sahney S, Ettenger R. Treatment of recurrent focal segmental glomerulosclerosis with high-dose cyclosporine A and plasmapheresis. Transpl Proc. 1993;25(1 Pt 2):1345–6.

49. Nathanson S, Cochat P, André J-L, Guyot C, Loirat C, Nivet H, et al. Recurrence of nephrotic syndrome after renal transplantation: influence of increased immunosuppression. Pediatr Nephrol. Springer-Verlag; 2005;20(12):1801–4.

50. Noorlander I, Hesselink DA, Wabbijn M, Betjes MGH. High cut-off haemodialysis induces remission of recurrent idiopathic focal segmental glomerulosclerosis after renal transplantation but is no alternative to plasmapheresis. NDT Plus. Oxford University Press; 2011;4(5):321–3.

51. Novo, R; Lahoche-Manucci, A; Foulard, M; Dehennault M. Treatment of nephrotic syndrome recurrence after renal transplantation in children: the experience of one center in the north of France. Pediatr Nephrol. 2011/05/24 ed. 2011. p. 1612.

52. Ohta T, Kawaguchi H, Hattori M, Komatsu Y, Akioka Y, Nagata M, et al. Effect of pre- and postoperative plasmapheresis on posttransplant recurrence of focal segmental glomerulosclerosis in children. Transplantation. 2001;71(5):628–33.

53. Okano K, Sugimoto H, Jinnai H, Iwasaki T, Takano M, Tsukada M, et al. Flowcytometric analysis of lymphocytapheresis in a patient with recurrent FSGS after renal transplant. Intern Med. 2011;50(24):3009–12.

54. Otsubo S, Tanabe K, Shinmura H, Ishikawa N, Tokumoto T, Hattori M, et al. Effect of post-transplant double filtration plasmapheresis on recurrent focal and segmental glomerulosclerosis in renal transplant recipients. Ther Apher Dial. 2004;8(4):299–304.

55. Pardon A, Audard V, Caillard S, Moulin B, Desvaux D, Bentaarit B, et al. Risk factors and outcome of focal and segmental glomerulosclerosis recurrence in adult renal transplant recipients. Nephrol Dial Transpl. 2005/12/21 ed. 2006;21(4):1053–9.

56. Pinto viola M. Plasmapheresis and Rituximab for Recurrent Posttransplant Focal Segmental Glomerulosclerosis. A Case Report. Pediatr Transpl. 2009/04/16 ed. 2009;13 Suppl 1:85.

57. Ponikvar R, Bren A, Kandus A, Buturovic Ponikvar J. Treatment of recurrence of focal segmental glomerular sclerosis after kidney transplantation with plasma exchange and immunoadsorption. Transpl Proc. 2001;33(7-8):3365–7.

58. Ponticelli C, Campise M, Tarantino A. The different patterns of response to plasmapheresis of recurrent focal and segmental glomerulosclerosis. Transpl Proc. 2002;34(8):3069–71.

59. Pradhan M, Petro J, Palmer J, Meyers K, Baluarte HJ. Early use of plasmapheresis for recurrent post-transplant FSGS. Pediatr Nephrol. 2003;18(9):934–8.

60. Raafat RH, Kalia A, Travis LB, Diven SC. High-dose oral cyclosporin therapy for recurrent focal segmental glomerulosclerosis in children. Am J Kidney Dis. 2004;44(1):50–6.

61. Ruhi, Caglar; Yilmaz, VT; Akpinar, MY; Akin, Olgun; Kocak, Huseyin; Suleymanlar G. The effect of plasmaferesis on focal segmental glomerulosclerosis relapse and graft survival in kidney transplantation. European Renal Association: Transplantation - clinical research 1. 2010. p. Sa677.

62. Saeed B, Mazloum H, Askar M. Spontaneous remission of post-transplant recurrent focal and segmental glomerulosclerosis. Saudi J Kidney Dis Transpl. 2011;22(6):1219–22.

63. Saleem MA, Ramanan A V, Rees L. Recurrent focal segmental glomerulosclerosis in grafts treated with plasma exchange and increased immunosuppression. Pediatr Nephrol. 2000;14(5):361–4.

64. Schachter AD, Harmon WE. Single-center analysis of early recurrence of nephrotic syndrome following renal transplantation in children. Pediatr Transplant. 2001;5(6):406–9.

65. Schachter ME, Monahan M, Radhakrishnan J, Crew J, Pollak M, Ratner L, et al. Recurrent focal segmental glomerulosclerosis in the renal allograft: single center experience in the era of modern immunosuppression. Clin Nephrol. 2010;74(3):173–81.

66. Sethna C, Benchimol C, Hotchkiss H, Frank R, Infante L, Vento S, et al. Treatment of recurrent focal segmental glomerulosclerosis in pediatric kidney transplant recipients: effect of rituximab. J Transpl. 2011;2011:389542.

67. Shariatmadar S, Noto TA. Therapeutic plasma exchange in recurrent focal segmental glomerulosclerosis following transplantation. J Clin Apher. Wiley Subscription Services, Inc., A Wiley Company; 2002;17(2):78–83.

68. Shetty R. Successful Treatment of Rapidly Recurrent FSGS with Plasmapheresis and Rituximab Rajesh. Am J Transpl. 2011/01/19 ed. 2011;11 Suppl 1:71.

69. Singh D, West K, Kamal K, Gupta R, Belitsky P, Kiberd B, et al. Early recurrence of primary focal segmental glomerulosclerosis in an older cadaveric renal allograft recipient resistant to plasmapheresis. Urology. 2006;67(1):200.

70. Stewart ZA, Shetty R, Nair R, Reed AI, Brophy PD. Case report: successful treatment of recurrent focal segmental glomerulosclerosis with a novel rituximab regimen. Transpl Proc. 2011;43(10):3994–6.

71. Torretta L, Perotti C, Costamagna L, Tarantino A, Salvaneschi L. Usefulness of plasma exchange in recurrent nephrotic syndrome following renal transplant. Artif Organs. 1995/01/01 ed. 1995;19(1):96–8.

72. Tsagalis G, Psimenou E, Nakopoulou L, Laggouranis A. Combination treatment with plasmapheresis and rituximab for recurrent focal segmental glomerulosclerosis after renal transplantation. Artif Organs. 2011;35(4):420–5.

73. Valdivia P, Gonzalez Roncero F, Gentil MA, Jimenez F, Algarra G, Pereira P, et al. Plasmapheresis for the prophylaxis and treatment of recurrent focal segmental glomerulosclerosis following renal transplant. Transpl Proc. 2005;37(3):1473–4.

74. Vyas, Shefali; Roberti I. Highly successful therapy of early FSGS recurrence post kidney transplant (txp): a single center experience. Pediatr Transplant. Blackwell Publishing Ltd; 2011;15:9.

75. Wuhl E, Fydryk J, Wiesel M, Mehls O, Schaefer F, Scharer K. Impact of recurrent nephrotic syndrome after renal transplantation in young patients. Pediatr Nephrol. 1998/10/07 ed. 1998;12(7):529–33.

76. Yabu JM, Ho B, Scandling JD, Vincenti F. Rituximab failed to improve nephrotic syndrome in renal transplant patients with recurrent focal segmental glomerulosclerosis. Am J Transpl. 2007/11/06 ed. 2008;8(1):222–7.

77. Zimmerman SW. Plasmapheresis and dipyridamole for recurrent focal glomerular sclerosis. Nephron. 1985;40(2):241–5.

Citations of Excluded Studies

1. Akioka K, Okamoto M, Ushigome H, Kadotani Y, Ogino S, Higuchi A, et al.: Recurrence of focal glomerulosclerosis in post-renal transplant recipients: report of two cases. Transplant Proc 2004 Sep;36:2167–2168.

2. Basri N, Shaikh I, Shaheen FAM: Plasmapheresis in renal transplant patients: a single-center experience. Transplant Proc 2003 Nov;35:2759–2760.

3. Bertelli R, Ginevri F, Caridi G, Dagnino M, Sandrini S, Di Duca M, et al.: Recurrence of focal segmental glomerulosclerosis after renal transplantation in patients with mutations of podocin. Am J Kidney Dis 2003 Jun;41:1314–1321.

4. Bruneau S, Dantal J: New insights into the pathophysiology of idiopathic nephrotic syndrome. Clin Immunol 2009 Oct;133:13–21.

5. Carl S, Wiesel M, Wuhl E, Mehls O, Schaefer F, Staehler G: Outcome of children and adolescents with recurrent nephrotic syndrome and focal segmental glomerulosclerosis after renal transplantation. Transplant Proc 1997 Nov;29:2795–2796.

6. Dantal J, Godfrin Y, Koll R, Perretto S, Naulet J, Bouhours JF, et al.: Antihuman immunoglobulin affinity immunoadsorption strongly decreases proteinuria in patients with relapsing nephrotic syndrome. J Am Soc Nephrol 1998 Sep;9:1709–1715.

7. Fornoni A, Sageshima J, Wei C, Merscher-Gomez S, Aguillon-Prada R, Jauregui AN, et al.: Rituximab targets podocytes in recurrent focal segmental glomerulosclerosis. Sci Transl Med 2011 Jun;3:85ra46.

8. Garcia V, Abbud-Filho M, Keitel E, Neumann J, Goldani JC: Recurrent focal glomerulosclerosis in renal allografts. Transplant Proc 1995 Feb;27:1084–1085.

9. Ghiggeri GM, Aucella F, Caridi G, Bisceglia L, Ghio L, Gigante M, et al.: Posttransplant recurrence of proteinuria in a case of focal segmental glomerulosclerosis associated with WT1 mutation. Am J Transplant 2006 Sep;6:2208–2211.

10. Hanevold CD, Greenbaum LA: Nephrotic syndrome after conversion to alternate day steroids in two children with a history of recurrent FSGS. Pediatr Transplant 2003 Oct;7:395–399.

11. Holgado R, Del Castillo D, Mazuecos A, Garcia T, Soriano S, Perez R, et al.: Long-term outcome of focal segmental glomerulosclerosis after renal transplantation. Transplant Proc 1999 Sep;31:2304–2305.

12. Ingulli E, Tejani A: Incidence, treatment, and outcome of recurrent focal segmental glomerulosclerosis posttransplantation in 42 allografts in children--a single-center experience. Transplantation 1991 Feb;51:401–405.

13. Itami N, Akutsu Y, Tochimaru H, Takekoshi Y, Seki T, Togashi M: Recurrent Steroid-Resistant Nephrotic Syndrome. Transplantation 1990;49.

14. Jain S, John E, Setty S, Benedetti E: Early recurrence of primary disease after pediatric renal transplantation: two case reports and a review of the literature. Pediatr Transplant 2007 Mar;11:217–221.

15. Jhaveri KD, Naber TH, Wang X, Molmenti E, Bhaskaran M, Boctor FN, et al.: Treatment of recurrent focal segmental glomerular sclerosis posttransplant with a multimodal approach including high-galactose diet and oral galactose supplementation. Transplantation 2011 Mar;91:e35–6.

16. Meyer TN, Thaiss F, Stahl RAK: Immediate versus long-term effect of rituximab in recurrent focal segmental glomerulosclerosis. Transpl Int 2008 Nov 1;21:1102.

17. Mizuiri S, Kawamura T, Miyagi M, Arai K, Sakai K, Aikawa A, et al.: Post-transplant early recurrent proteinuria in patients with focal glomerulosclerosis--angiotensin II immunostaining and treatment outcome. Clin Transplant 2005;19 Suppl 1:12–19.

18. Oetliker OH, Zimmermann A, Bianchetti MG: Treatment of recurrent idiopathic nephrotic syndrome after transplantation using plasmaphaeresis and intensified immunosuppression over 2 months. Pediatr Nephrol7:508.

19. Ohta T, Sakano T: Post-transplant recurrence of focal segmental glomerulosclerosis. Kidney Int 2016 Feb 8;59:2374.

20. Ponticelli C, Passerini P: Alternative treatments for focal and segmental glomerular sclerosis. Clin Nephrol 2001 May;55:345–348.

21. Stephanian E, Matas AJ, Mauer SM, Chavers B, Nevins T, Kashtan C, et al.: Recurrence of disease in patients retransplanted for focal segmental glomerulosclerosis. Transplantation 1992 Apr;53:755–757.

22. Trachtman H, Futterweit S, Singhal PC, Franki N, Sharma M, Sharma R, et al.: Circulating factor in patients with recurrent focal segmental glomerulosclerosis postrenal transplantation inhibits expression of inducible nitric oxide synthase and nitric oxide production by cultured rat mesangial cells. J Investig Med 1999;47:114–120.

23. Weber S, Tönshoff B: Recurrence of Focal-Segmental Glomerulosclerosis in Children after Renal Transplantation: Clinical and Genetic Aspects. Transplantation 2005;80.

24. Yakupoglu U, Baranowska-Daca E, Suki WN, Truong LD: New aspects of posttransplant nephrotic syndrome: clinicopathologic correlations with outcomes. Transplant Proc 2004;36:139–143.

25. Williams ME, Hamdan A: Comparative effects of centrifuge-and membrane-based pheresis treatments on proteinuria in a patient with recurrent kidney transplant focal glomerulosclerosis. J Clin Apher 2012; 27:36.

26. Baskin E;Bayrakci U.S.;Ozdemir H;Demirhan B;Cengiz N;Karakayali H;Sevmis S;Haberal: The outcome of renal transplantation in children with FSGS. Pediatr Transplant 2009;13:46–156.

27. Couloures K, Pepkowitz SH, Goldfinger D, Kamil ES, Puliyanda DP: Preventing recurrence of focal segmental glomerulosclerosis following renal transplantation: A case report. Pediatr Transplant10:962–965.

28. Dantal J, Testa A, Bigot E, Soulillou JP: Effects of plasma-protein A immunoadsorption on idiopathic nephrotic syndrome recurring after renal transplantation. Ann Med Interne (Paris) 1992;143 Suppl :48–51.

29. Rahamimov R, Mor M, Eizner S, Yussim A, Solomonov E, Nesher E, et al.: Recurrence of FSGS among adult transplant recipients: A longitudinal study. Transpl Int 2011 Sep 1;24:134.

30. Jason H. Kang, MD, Lauren Jefferies-Zeitler, RN, PhillipJ. DeChristopher, MD P: Explosive Diarrhea As a novel complication Of Therapeutic Plasmaexchange (TPE) In Patients Treated For Recurrent Focal Segmental Glomerulosclerosis (FSGS). J Clin Apher 2011 Jan 1;26:64.

31. Kawaguchi H, Hattori M, Kubota R, Ito K, Takahashi K, Agishi T, et al: Plasma exchange for recurrent nephrotic syndrome due to focal segmental glomerulosclerosis in pediatric renal transplants. Artif Organs 1992 Dec 1;16:655.

32. Miyauchi Y, Shirakawa H, Shimizu T, Omoto K, Ishida H, Tanabe K: Excellent outcomes of rituximab administration plus plasmapheresis as prophylactic treatment prior to kidney transplantation in patients with focal segmental glomerulosclerosis. Am J Transplant 2011 Apr 1;11:427.

33. Marisol M, B Restrepo JM CG, Eliana: Outcome of kidney transplantation in children with nephrotic syndrome-FSGS using novel treatment: Plasmapheresis-PL- and high dose of cyclosporine -CyA. Pediatr Transplant 2011 Aug 1;15:55.

34. Al-Solaiman Y, DeMattos A, Marques B, Kumar V: Degree of proteinuria at completion of plasma exchange is a predictor of allograft loss in recurrent FSGS. Am J Transplant 2012 May 1;12:378.

35. Somers M, Baum M: Pre-transplant conditioning with plasmapheresis and cyclosporine infusion reduces recurrence of focal segmental glomerulosclerosis (fsgs) in children. Pediatr Transplant 2009;13:96.

36. Munoz J, Sanchez M, Perez-Garcia R, Anaya F, Valderrabano F: Recurrent focal glomerulosclerosis in renal transplants proteinuria relapsing following plasma exchange. Clin Nephrol 1985 Oct;24:213–214.

37. Savin VJ, Sharma R, Sharma M, McCarthy ET, Swan SK, Ellis E, et al.: Circulating factor associated with increased glomerular permeability to albumin in recurrent focal segmental glomerulosclerosis. N Engl J Med 1996 Apr;334:878–883.

38. Y, Willassen: Reduction of Proteinuria Following Plasma-Exchange in Recurrent Renal-Allograft Focal Glomerulosclerosis. Kidney Int 1982 Feb 20;21:669.
